# Creating and validating photorealistic AI-generated facial expression stimuli for emotion research

**DOI:** 10.3758/s13428-026-03156-0

**Published:** 2026-09-22

**Authors:** Shlomo Hareli, Shlomo David

**Affiliations:** The Laboratory for the Study of Social Perception of Emotions, School of Business Administration, The Herta & Paul Amir Faculty of Social Sciences, University of Haifa, 199 Aba Khoushy Ave., Mount Carmel, Israel

**Keywords:** **A**rtificial intelligence, Facial expressions, Emotion perception, Stimulus validation, Body weight

## Abstract

The development of AI-generated facial expression stimuli offers researchers unprecedented control over stimulus characteristics while maintaining photorealistic quality. However, rigorous validation methodologies are essential to ensure these stimuli function effectively in behavioral research. This paper presents a comprehensive three-study validation framework for AI-generated facial expression stimuli that systematically varied body weight and emotional expressions. Study 1 validated 72 stimuli across three emotions (happiness, sadness, neutrality) and three weight categories using both computational and human validation approaches. Study 2 extended this framework to include anger as a fourth emotion while controlling for individual facial features through same-character methodology. Study 2b further assessed valence, intensity, naturalness, authenticity, and non-focal emotion ratings of the same stimuli from Study 2. Across studies, the stimuli demonstrated successful emotion and weight manipulations, with AI-generated images achieving photorealistic quality that participants could not reliably distinguish from real photographs. Computational validation showed high intended-emotion likelihood in Py-Feat, while human validation confirmed appropriate perception of the target emotions, body weight, realism, age, and broader expression-quality dimensions. This dual validation approach—combining automated computational assessment with human behavioral validation—provides a robust framework for ensuring AI-generated stimuli meet research standards. The methodology offers significant advantages for behavioral research, enabling precise control over stimulus characteristics while maintaining ecological validity and addressing ethical considerations in stimulus development.

Emotion perception plays a crucial role in social communication and interpersonal relationships, enabling individuals to understand others’ mental states, infer their characteristics, and interpret situational contexts. This process facilitates adaptive responses to the situation at hand (Hareli & Hess, 2010; Hess & Hareli, 2016; Parkinson, 1996; Van Kleef, 2009; van Kleef et al., 2004). Accordingly, extensive research has examined social perception of emotions across multiple domains, including social and clinical psychology, neuroscience, and psychiatry (Brooks & Freeman, 2018; Collin et al., 2013; Kring & Elis, 2013; Schirmer & Adolphs, 2017).

Images depicting emotional facial expressions represent a central methodological tool in emotion perception research (Adolphs, 2002; Barrett et al., 2019; Dawel et al., 2022; Ekman & Friesen, 1976; Russell, 1994; Wallbott, 1998). The methodology involves creating facial expression stimuli with systematic control over the emotions displayed, followed by validation to ensure they reliably convey the intended emotions to observers. These stimuli are then used in research studying emotion perception and its underlying mechanisms. This paper presents a novel method leveraging generative AI to create photorealistic facial expression stimuli and a rigorous procedure for their validation, highlighting the potential advantages for emotion perception studies.

## Current approaches and their limitations

Traditionally, researchers have relied primarily on photographic datasets of human actors expressing emotions. The early POFA database (Pictures of Facial Affect; Ekman & Friesen, 1976) focused on the six basic emotions identified by Ekman (1992). Other subsequent datasets also focused on basic emotions, including KDEF (Karolinska Directed Emotional Faces; Lundqvist et al., 1998) and FACES (Ebner et al., 2010). Some datasets extend beyond the foundational set, such as RaFD (Radboud Faces Database; Langner et al., 2010), which incorporates contempt, and ADFES (Amsterdam Dynamic Facial Expression Set; Van der Schalk et al., 2011), which also features contempt, pride, and embarrassment. Some datasets also vary in characteristics such as age of models (e.g., FACES and RaFD), ethnicity (e.g., Chicago Face Database, Ma et al., 2015; NimStim, Tottenham et al., 2009; ADFES), expression parameters such as head pose or gaze direction (e.g., KDEF and RaFD), and whether expressions are posed versus spontaneous (e.g., DISFA, Mavadati et al., 2013).

While this approach has proved fruitful, it also has some limitations. Controlling actors to produce precise emotional expressions is challenging, and existing datasets often lack the diversity of social identities and appearance features found in real-world populations (Barrett et al., 2019; Barrett et al., 2011). To address the need for greater experimental control and broader identity variation, researchers have developed sophisticated computational approaches including 3D parametric models (3DMMs; Blanz & Vetter, 1999; Egger et al., 2020) and FACS-based animation software (e.g., FACSGen, Roesch et al., 2011). These computational methods can generate diverse identities and systematic variation in appearance characteristics, but they face their own persistent challenges: FACS-based tools and 3D modelling programs require expensive specialized software and extensive technical training, often taking months to master. Furthermore, even sophisticated 3D approaches often struggle with the uncanny valley effect, producing stimuli that participants perceive as artificial or unsettling (Dawel et al., 2022; Wang et al., 2015). Evidence also suggests differential responses to virtual versus human faces, potentially affecting research validity (Balas & Pacella, 2015; Katsyri et al., 2020; Miller et al., 2022).

## AI-generated stimuli: A promising alternative

Recent advances in generative AI offer a promising alternative approach that potentially addresses some of these limitations. AI-generated stimuli can provide photorealistic quality without being constrained by existing photographic datasets or suffering from uncanny valley effects while offering systematic control over demographic variables, facial expressions, and identity characteristics (Han et al., 2022; Miller et al., 2023; Nightingale & Farid, 2022). A recent example is the work by Solis-Arrazola et al. (2025), who used Midjourney-generated images of children’s emotional expressions, together with real child-face databases, to train and validate emotion-recognition algorithms based on facial muscle activation. This approach eliminates the need for expensive specialized software while enabling reproducible, well-documented methodologies. Generative AI used to create stimuli also allows for a large variety of model identities, including those depicting characteristics that might be more ethically problematic to portray using real people’s photographs. Additionally, AI generation enables the creation of stimuli specifically designed for experimental flexibility such as generating images with standardized green backgrounds that facilitate green screen methodology, allowing researchers to systematically replace backgrounds in post-processing to study how different environmental contexts influence emotion perception.

This contextual flexibility offered by AI-generated stimuli is particularly valuable given the growing recognition that emotional expression perception is heavily influenced by situational and environmental factors (Aviezer et al., 2017; Barrett et al., 2019; Hassin et al., 2013; Hess & Hareli, 2014).

Across all these approaches—from traditional photographic datasets to sophisticated computational methods to emerging AI techniques—researchers face a fundamental challenge: no single approach to creating emotional expression stimuli can fully overcome the inherent tension between experimental control and ecological validity. Any method that increases control necessarily sacrifices some aspect of naturalistic emotional expression and/or character realism, while approaches that prioritize ecological validity inevitably compromise experimental precision. However, generative AI seems to relax this inherent tension by enabling the creation of stimuli representing a much larger and more diverse set of model identities than other methods while maintaining photorealistic appearance and control. Unlike computational approaches that resort to avatar-like representations for diverse or less common populations (which compromise realism), or photographic datasets that are constrained by available actors or existing images and/or ethical concerns, AI can generate realistic-appearing faces across a broad spectrum of identities without sacrificing either experimental control or visual authenticity and doing so in a more ethical way.

The goal of the present work was not simply to add another facial-expression stimulus set, but to demonstrate a reproducible method for generating controlled, photorealistic stimuli when existing databases do not provide the required combination of emotion, identity, and appearance features. Existing databases are invaluable, but they are limited by the available actors, expressions, identities, body types, and image formats. Generative AI can help researchers create stimuli with appearance features that may be rare, underrepresented, difficult to recruit, or ethically sensitive to portray using photographs of real people, while maintaining control over features such as background, clothing, pose, and character identity. The present approach therefore complements existing databases by allowing researchers to create and validate stimuli tailored to specific experimental requirements.

### The present methodology

The present paper describes a method for using generative AI to create realistic images of facial expressions of emotions and a procedure for validating these stimuli not just in terms of the expressions shown but also other relevant and critical characteristics such as realism and figure identity. Presented are two resulting datasets, with identity of figures controlled and manipulated. The methods make use of the open-source platform ComfyUI (ComfyAnonymous, 2024), which enables researchers to create highly realistic facial expression stimuli through systematic, reproducible workflows. An important component of this approach is the development of custom-trained LoRA (Low-Rank Adaptation; Hu et al., 2021) models that enable precise control over specific facial expressions while maintaining photorealistic quality. This sophisticated multi-workflow methodology demonstrates how ComfyUI can serve as a flexible platform for implementing advanced stimulus generation pipelines that integrate custom LoRA training, identity preservation techniques (PuLID; Lin et al., 2024), face-swapping algorithms, and rigorous validation protocols.

To demonstrate the methodology’s effectiveness and versatility, we present three validation studies using stimuli depicting individuals of different body weights expressing various emotions. Body weight manipulation serves as an ideal test case for validating our AI generation methodology because it: (1) involves systematic changes to facial morphology through features like facial width-to-height ratio (Coetzee et al., 2010), (2) demonstrates the methodology’s ability to control characteristics that naturally covary with multiple facial features, (3) can be validated through both computational metrics and human perception, and (4) raises ethical concerns when using real individuals’ photographs or recruiting actors whose body weight may be stigmatized. While existing databases feature individuals with primarily “average” facial features (Barrett et al., 2019; Barrett et al., 2011) and minimal morphological variation (Martin et al., 2024; Matsumoto et al., 1999), our methodology enables systematic manipulation of such characteristics while maintaining experimental control. Here we focus specifically on the stimulus creation, computational validation, and human validation procedures to establish the methodological framework. The substantive behavioral findings about how body weight influences emotion perception and social judgments are reported separately (Hareli & David, n.d.).

## Study 1

### Method

#### Stimulus creation and computational validation

Stimulus generation utilized the Flux.1-dev diffusion model (Black Forest Labs, 2024) enhanced with Low-Rank Adaptation fine-tuning (Hu et al., 2021) for expression-specific control. We generated 72 images depicting individuals from the chest up against a green background using ComfyUI (ComfyAnonymous, 2024), an advanced workflow-based platform designed for creating highly realistic and detailed visual content through systematic, reproducible pipelines. Custom LoRA models were trained using FluxGym (Cocktailpeanut, 2024) on subsets of the FACES database (Ebner et al., 2010). All workflows were implemented in ComfyUI with the expression editor (PowerHouseMan, 2024) used for iterative refinement of expressions until validation criteria were satisfied. All supplementary materials are openly available on OSF, including the image datasets, study data files, codebooks, analysis scripts, validation scripts, workflow screenshots, LoRA models, software attribution, computational-validation outputs, and complete workflow specifications at : https://osf.io/mbg4j/?view_only=a0c0512fa2b04cc19c335cc2bd299017.

#### Custom LoRA training for expression control

To ensure accurate and reliable representation of specific emotions across genders, a LoRA (Low-Rank Adaptation) technique was utilized within the workflow. Preliminary testing revealed that elaborate prompts alone with the base Flux1-dev model produced inconsistent emotional expressions, with Py-Feat classification confidence for the intended emotion falling below acceptable thresholds for systematic research use. LoRA allows for fine-tuning of pre-trained models to improve their performance on specialized tasks. The LoRA models were trained using FluxGym (Cocktailpeanut, 2024), a platform for adapting AI models to specific datasets and conditions. The training involved the FACES database (Ebner et al., 2010), which contains high-quality images of validated facial expressions. This database was used with explicit permission from its creators for LoRA training purposes. Specifically, for each combination of emotion (sadness, happiness, neutrality) and gender (man, woman), 15 images were randomly selected from the appropriate subset of FACES database. Each training image was accompanied by descriptive captions (e.g., “a young man expressing anger,” “a young woman expressing happiness”) that were initially generated automatically by FluxGym and then manually reviewed and modified as needed to ensure accuracy. Additionally, specific keywords were assigned to each emotion–gender combination (e.g., “angry man,” “happy woman,” “neutral man”) to serve as class tokens during training and subsequently as trigger words in generation prompts to activate the corresponding LoRA models.

##### LoRA training parameters

All models were trained using LoRA (Low-Rank Adaptation) with the following configuration: learning rate of 8e−4, 16 epochs, batch size of 1, and input resolution of 512×512 pixels. The LoRA implementation used a rank of 4, targeting single transformer blocks, and employed the Adafactor optimizer with a constant warmup learning rate scheduler. Training optimization included mixed precision (bf16) and gradient checkpointing to enhance memory efficiency. Each emotion–gender combination model was trained on 15 unique images, with each image repeated 10 times per epoch, resulting in 150 effective training samples per epoch and 2,400 total training steps per model.

Model checkpoints were saved every four epochs, and the final workflows utilized the model checkpoint from the 16th epoch. All training used a fixed seed of 42 to ensure reproducibility. Training was conducted on an NVIDIA RTX 4000 Ada Generation Laptop GPU with 12 GB VRAM. Each emotion–gender combination required approximately 6 h of training time, resulting in a total training duration of approximately 36 h for all six models.

#### Image generation process: Systematic prompt structure

Generation prompts followed a standardized format: “A hyper-realistic photo of a young Caucasian [body weight descriptor:2.9] [gender]. Light green background, rim lighting, studio lighting, dslr, ultra quality, sharp focus, (navy blue smooth one-piece short high-neck T_shirt:1.8), (facing the camera:2), UHD, 16K. (Hands down alongside the body:1.95), ([chest/waist] up:2), (top of head seen:2), clean arms, [emotion] [gender], [varying facial characteristics].” Numbers following colons (e.g., “:1.8,” “:2”) indicate attention weights assigned to specific prompt elements, with higher values emphasizing those characteristics during generation.

The body weight descriptors varied systematically across conditions (thin/middle weight/morbidly obese),^1^ while emotional expressions (sad/happy/neutral) and distinctive facial features (hair color, eye color, facial structure) were also changed in the prompt across generations to create diverse identities within each condition.

##### Computational validation

All generated images underwent validation using Py-Feat (Cheong et al., 2023), a Python library designed for facial expression analysis. We established a strict criterion that the expression in the image must have at least a 95% likelihood of being the desired emotion according to Py-Feat’s emotion detection algorithms.

#### Automated validation process

To streamline the validation workflow, we developed a custom Python script that automates the Py-Feat analysis process. The script prompts users to select a directory containing images, processes all image files using Py-Feat’s facial expression detection, and generates a comprehensive HTML report displaying each image alongside detailed emotion confidence scores and Action Unit activations. Images that did not meet the 95% intended-emotion likelihood criterion were edited using the Expression Editor in ComfyUI (PowerHouseMan, 2024)—a node-based tool that allows fine-grained adjustment of facial expressions in generated images—through an iterative refinement process until validation criteria were satisfied. This automated approach ensures consistent validation procedures and facilitates efficient processing of large image sets. The validation script, detailed usage instructions, example HTML output, and complete validation results for all dataset images in Excel format are available in the supplementary materials in the OSF.

##### Final dataset

**Fig. 1:**
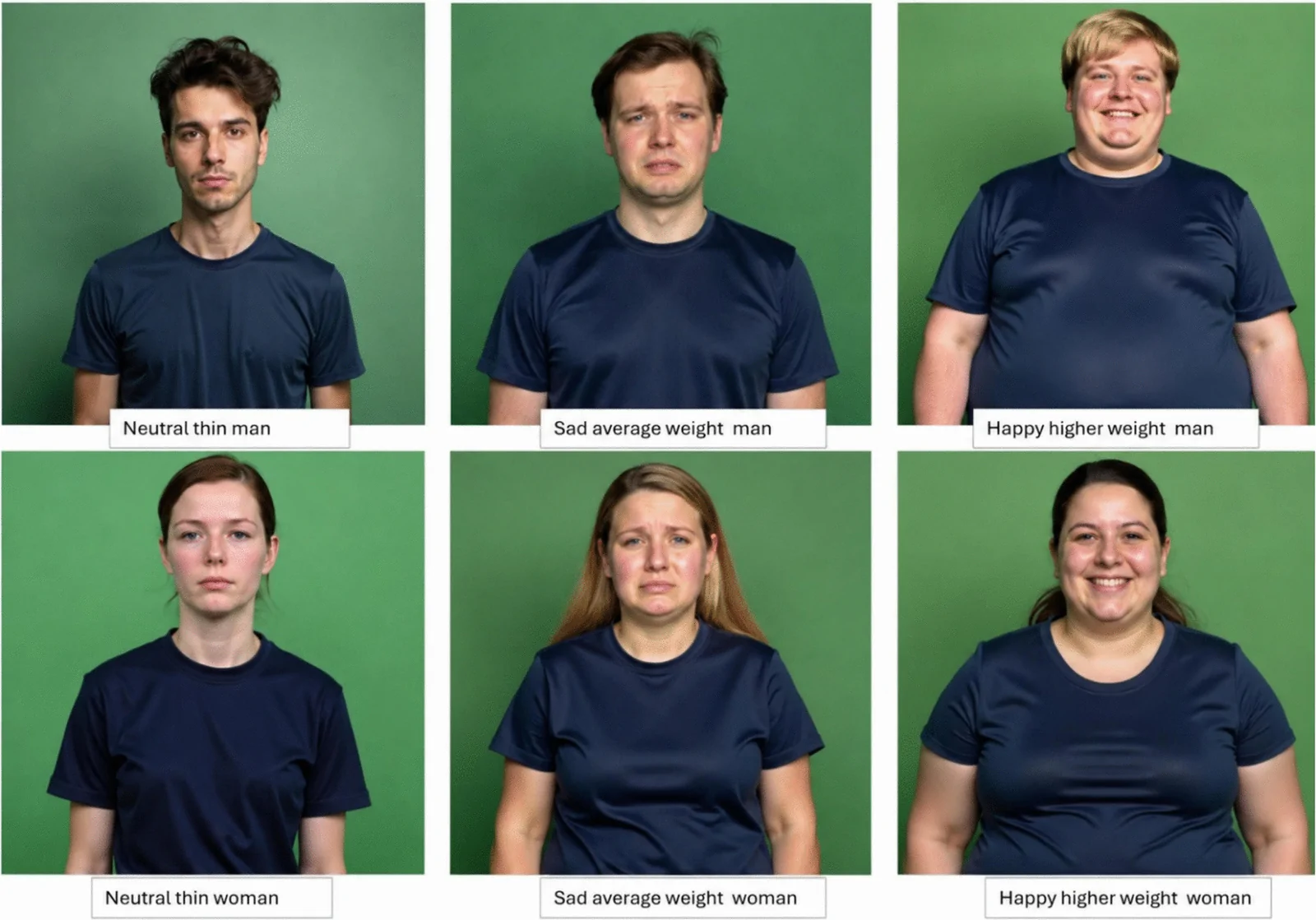
Examples of AI-generated stimuli illustrating emotional expressions and body types – Study 1

Following the validation and refinement process, the final dataset comprised 72 images systematically distributed as follows: (1) gender: 36 men and 36 women; (2) body weight categories: thin, average weight, and higher weight; (3) emotional expressions: happiness, sadness, neutrality. This yielded four individuals per gender–weight–expression combination. Py-Feat analysis of the final dataset yielded a mean intended-emotion likelihood score of *M* = 98.38% (*SD* = 1.54%), indicating high computational classification confidence for the intended emotion categories. Examples of images from this dataset appear in Fig. 1.

### Human validation

#### Study goals

Study 1 was designed to validate the computationally pre-validated AI-generated stimuli with human participants. While the stimuli had already undergone computational validation using Py-Feat analysis to ensure 95% Py-Feat classification confidence for the intended emotion, this study assessed whether human observers would perceive the stimuli as intended. Specifically, we tested whether participants would (1) correctly identify the intended emotions and body weight categories, and (2) perceive the images as photorealistic rather than AI-generated. We also collected (3) age ratings to examine whether any perceived age differences across body-weight and/or emotion conditions aligned with established research findings (see, e.g., Han et al., 1999; Hass et al., 2016; Henderson et al., 2016; Liu & Feng, 2023; Voelkle et al., 2012) or instead indicated problematic confounds. For instance, if individuals with higher body weight appeared older, these results would be compared against existing research on weight and age perception to determine whether the pattern matched known real-world phenomena. This would enable us to further determine the degree to which these AI images match images of real people.

#### Participants

A total of 606 US participants (274 men, 319 women, and 13 who identified as other) with a mean age of 40 years (*SD* = 14.37) were recruited through Prolific Academic. Participants were paid £0.75 for their participation in the study, which had an estimated duration of 7 min. The only exclusion criterion was that participants had to have an approval rating of 90% or higher to ensure response quality.

#### Procedure and dependent measures

Participants viewed one AI-generated image and completed several validation assessments in a between-subjects design. Participants in this study saw only one photo; hence our goal was to recruit at least 30 participants per cell to assure adequate mean interrater reliability (Rosenthal, 2005).

##### Manipulation check

Participants rated each photo on the degree to which the person expressed sadness, happiness, and neutrality. Unless otherwise specified, all ratings were made on 7-point Likert scales ranging from 0 (not at all) to 6 (to a large extent). Participants were also asked to estimate the weight of individuals in photos using a scale ranging from 110 pounds to 250 pounds. This range encompasses a wide variety of body weights from low to high. Participants were informed that the average weight for women is 171 pounds and for men, 198 pounds. This information, along with the weight range, was sourced from the Centers for Disease Control and Prevention (n.d.).

##### Stimulus quality assessment

Another rating asked participants to estimate the likelihood that the person in the photo was a real person or an AI-generated image. Participants were informed that the image they saw was randomly selected from a mixed repository containing both real and AI-generated photos. The rating scale ranged from 0 (Definitely a real person) to 6 (Definitely an AI-generated photo), with the midpoint, 3, indicating “Can't decide.” This measure targets perceived realness, that is, the extent to which participants judged the image as depicting a real person rather than as AI-generated. It is conceptually distinct from the broader expression-quality dimensions introduced later in Study 2b, namely naturalness and authenticity, which assess properties of the depicted expression rather than perceived realness or AI detection. Finally, participants also rated the perceived age of the person in the photo on a scale ranging from 16 to 70 years.

#### Results

We conducted a series of 3 (emotion expression) × 3 (weight group) × 2 (gender) Type III analyses of variance (ANOVA) in RStudio (RStudio Team, 2019) using the car package for the ANOVA calculation (Fox & Weisberg, 2019), the effectsize package for effect size estimation (Ben-Shachar et al., 2020), and emmeans package for post hoc analyses, employing Tukey’s method for pairwise comparisons.

##### Perceived emotions

For all emotion expression ratings, a main effect of emotion expression emerged: *F*(2, 588) = 258.56, *p* < .001, ηp2 = .47; *F*(2, 588) = 736.71, *p* < .001, ηp2 = .71; and *F*(2, 588) = 318.80, *p* < .001, ηp2 = .48, for sadness, happiness, and neutrality, respectively. For happiness, the emotion × gender × weight interaction, *F*(4, 588) = 3.02, *p* = .018, ηp2 = .020, did not qualify the emotion manipulation; post hoc comparisons showed no significant differences among cells within the happy condition. For neutral, the emotion × weight interaction, *F*(4, 588) = 3.19, *p* = .013, ηp2 = .021, similarly did not qualify the manipulation; no post hoc comparison between weight levels within any single emotion condition reached significance (see Markdown of Study 1 in the OSF).

**Fig. 2:**
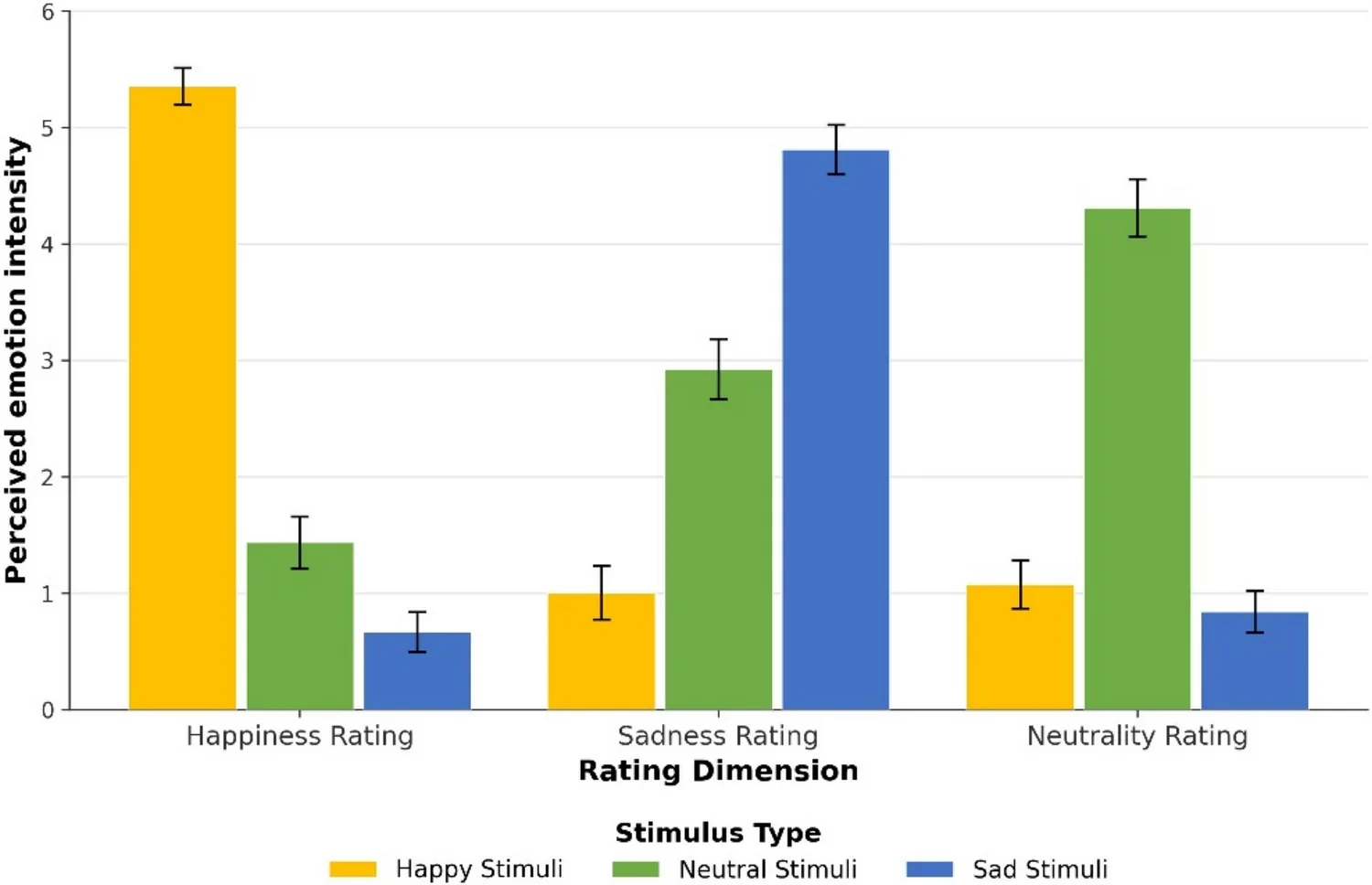
Effects of emotion expression condition on perceived happiness, sadness, and neutrality: means and confidence intervals – Study 1

As shown in Fig. 2, happiness was rated significantly happier than neutrality, which seemed happier than sadness. Sadness was rated significantly sadder compared to neutrality, which in turn was perceived as sadder than happiness. Finally, neutrality was perceived as significantly more neutral than both sadness and happiness, which were perceived as equally neutral. Overall, all emotion conditions were perceived as planned, across weight groups and gender.

##### Inter-rater reliability of perceived focal emotions

Inter-rater reliability of the emotion ratings was assessed using a one-way random-effects intraclass correlation (ICC(1,k); Shrout & Fleiss, 1979), treating image as the grouping factor. This approach follows the same logic used to validate the Radboud Faces Database (Langner et al., 2010). Reliability on the focal emotion rating was fair ICC(1,k) = .549, 95% CI [.349, .662], (Cicchetti, 1994), based on an average of 8.42 raters per image (range 2–17; 72 images, 606 ratings).

##### Perceived weight

A significant main effect of weight group, *F*(2, 588) = 493.11, *p* < .001, ηp2 = .63, and gender, *F*(1, 588) = 249.57, *p* < .001, ηp2 = .30, emerged. There were also significant interactions between gender and weight group, *F*(2, 588) = 10.51, *p* < .001, ηp2 = .03, and between emotion expression and weight group, *F*(4, 588) = 3.61, *p* = .006, ηp2 = .03. As intended, there was a clear, significant gradual increase in perceived weight (measured in pounds) from the thin-weight group (*M* = 160.60, *SD* = 29.20; 95% CI [156.65, 164.61]), through the average-weight group (*M* = 203.02, *SD* = 26.77; 95% CI [199.28, 206.76]), to the group identified as higher weight (*M* = 224.49, *SD* = 20.41; 95% CI [221.63, 227.35]). Additionally, men (*M* = 208.50, *SD* = 31.22; 95% CI [205.0, 212.17]) were perceived as having higher weight than women (*M* = 182.57, *SD* = 37.85; 95% CI [178.32, 186.82]), fitting actual typical differences in weight between the genders (Wells, 2007).

The interaction between gender and weight group partially qualified the main effect of gender as it resulted from the fact that higher-weight women and average-weight men did not differ in terms of perceived weight (see Markdown of Study 1 in the OSF). The interaction between emotion expression and weight group failed to show any significant effects in the post hoc analysis that did not reflect the main effect of weight group.

##### Perceived realness of images

Significant interactions between emotion expression and gender, *F*(2, 588) = 4.09, *p* = .017, ηp^2^ = .01, and between gender and weight group, *F*(2, 588) = 7.67, *p* < .001, ηp^2^ = .03, emerged. Post hoc analyses showed that neutral men (*M* = 2.80, *SD* = 1.96; 95% CI [2.41, 3.19]) and sad women (*M* = 2.82, *SD* = 2.04; 95% CI [2.42, 3.21]) were rated as equally and somewhat less real than the happy women (*M* = 1.87, *SD* = 1.82; 95% CI [1.52, 2.21]). The interaction between gender and weight group resulted from the fact that the higher-weight men (*M* = 3.25, *SD* = 2.08; 95% CI [2.82, 3.68]) were rated somewhat less real than all other conditions except for the thin women (*M* = 2.68, *SD* = 1.82; 95% CI [2.32, 3.03]). As can be seen, the condition rated as least resembling a human scored close to 3 on the scale, which corresponds to the “Can't decide” option. This implies that, despite some differences across conditions, none were clearly identified as AI-generated images. Image-level realness and validation ratings for each Study 1 stimulus are reported in Table 1 in the supplementary materials on OSF, allowing readers to identify the stimuli that were perceived as most and least real.

##### Perceived age

Main effects were found for emotion expression, *F*(2, 588) = 17.99, *p* < .001, ηp2 = .06, gender, *F*(1, 588) = 13.42, *p* < .001, ηp2 = .02, and weight group, *F*(2, 588) = 48.77, *p* < .001, ηp2 = .14. Happy persons appeared younger (*M* = 30.78, *SD* = 7.82; 95% CI [29.71, 31.86]) than sad (*M* = 34.67, *SD* = 8.46; 95% CI [33.52, 35.82]) and neutral persons (*M* = 33.81, *SD* = 8.66; 95% CI [32.59, 35.03]). Perceived age also increased systematically across weight groups: *M* = 29.00, *SD* = 7.41; 95% CI [27.99, 30.01] for thin, *M* = 34.22, *SD* = 7.75; 95% CI [33.14, 35.29] for average weight, and *M* = 36.26, *SD* = 8.54; 95% CI [35.07, 37.45] for higher weight. Additionally, women (*M* = 34.28, *SD* = 8.28; 95% CI [33.35, 35.20]) appeared somewhat older than men (*M* = 31.86, *SD* = 8.50; 95% CI [30.90, 32.83]). The two findings mentioned first align with previous findings from studies of real people, as it has been shown that happy expressions cause people to be perceived as younger (Hass et al., 2016; Voelkle et al., 2012). Also, facial and body morphology associated with higher weight, such as a larger lower face, wider jaw, and wider waist, particularly in individuals around the age of 30—the age group of the present stimuli—can contribute to perceptions of being older (Han et al., 1999; Henderson et al., 2016; Liu & Feng, 2023). Of most importance is the fact that the stimuli across conditions appeared to be of the same general age group.

#### Discussion

The validation results demonstrate that the AI-generated stimuli successfully functioned as intended across all critical dimensions. The correspondence between computational Py-Feat classification confidence and human ratings suggests that the automated validation procedure was useful for screening AI-generated facial expressions before human validation. Specifically, the clear pattern shown in Fig. 2 validates the effectiveness of our custom LoRA training approach. Each emotion achieved high ratings on its intended dimension while receiving appropriately low ratings on non-target emotions, indicating that the computational refinement process successfully created distinct, recognizable emotional expressions.

The perceived realness analysis revealed nuanced but reassuring patterns. While significant interactions emerged between emotion expression and gender, and between gender and weight group, these effects were subtle. Critically, however, even the condition rated as least resembling a human scored close to the “Can’t decide” option. This demonstrates that current AI generation can achieve photorealistic quality suitable for behavioral research across diverse conditions, consistent with recent findings by others (Han et al., 2022; Miller et al., 2023; Nightingale & Farid, 2022). This advancement addresses a major limitation of previous computational approaches that suffered from uncanny valley effects (Dawel et al., 2022; Wang et al., 2015).

The systematic perceived age and emotion differences across conditions aligned with established research, enhancing rather than compromising the stimuli’s validity. These patterns suggest the AI generation process captured realistic correlations between physical characteristics, age and emotion perception rather than creating artificial associations, enhancing the ecological validity of the stimuli.

The weight manipulation validation revealed both main effects and meaningful interactions. The clear gradual increase across weight categories confirmed successful manipulation. The gender × weight interaction revealed that higher-weight women and average-weight men did not differ in perceived weight, reflecting realistic population distributions (Wells, 2007). This again enhances the ecological validity of the stimuli.

Despite these successful validation outcomes, Study 1 had methodological limitations that warranted addressing. We used different characters per condition, a strategy that may have introduced potential facial feature confounds. Study 2 was designed to mitigate this limitation by employing the same AI-generated character across emotion conditions—a standard practice enhancing internal validity (cf. Ebner et al., 2010; Lundqvist et al., 1998; Van der Schalk et al., 2011). Additionally, Study 2 incorporated anger as a fourth emotion to further explore the usefulness and versatility of this AI generation method. The inclusion of anger allowed us to test the generalizability of the methodology across a broader range of emotional expressions.

## Study 2

### Method

#### Stimulus creation and computational validation

**Fig. 3:**
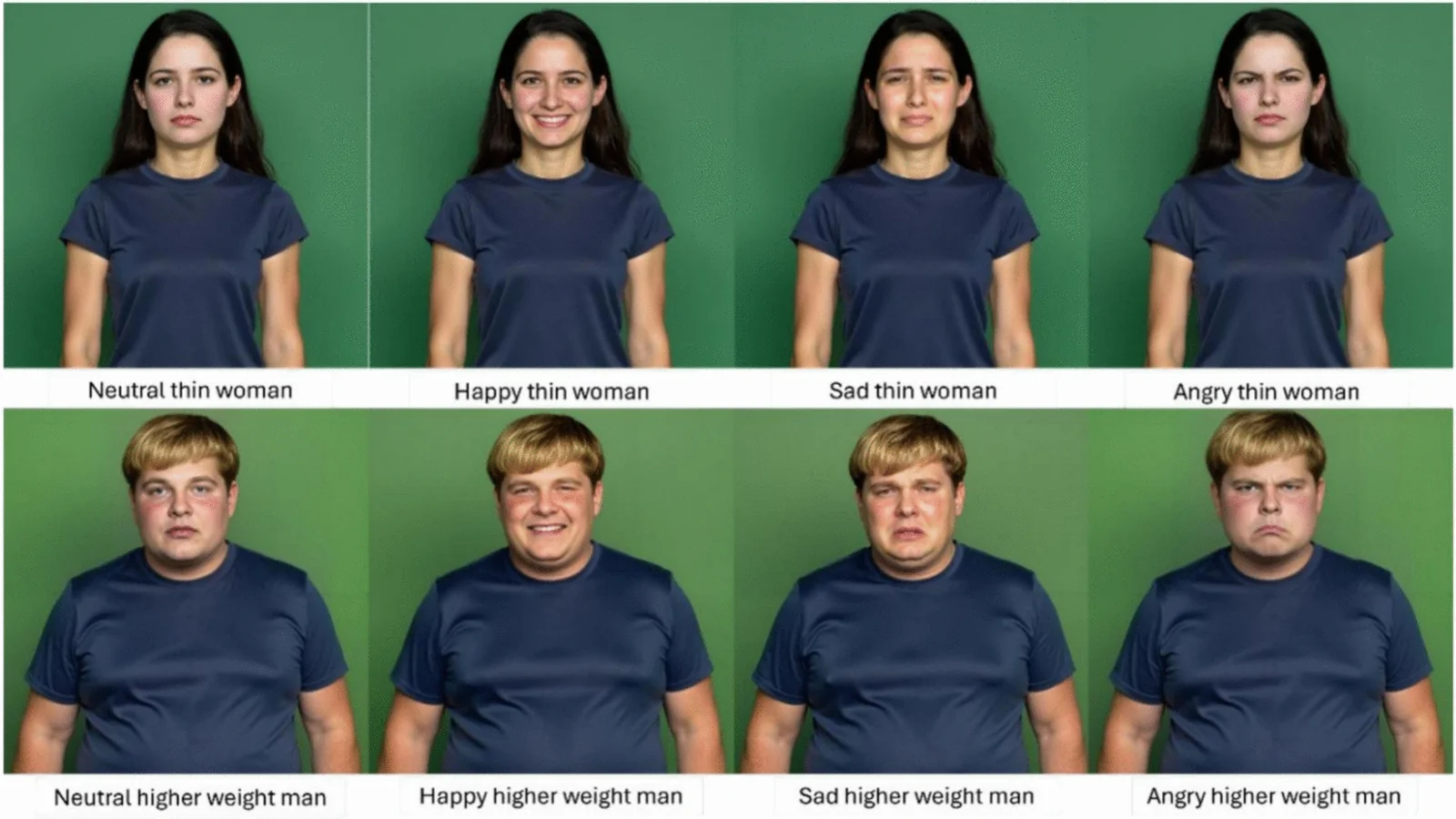
Examples of AI-generated stimuli illustrating emotional expressions and body types – Study 2

Study 2 employed the same core technologies as Study 1 (ComfyUI, Flux1-dev model, custom LoRA training using FACES database) but required additional workflows to maintain character identity across different emotional expressions. Using ComfyUI, 96 AI-generated images were created, featuring 12 men and 12 women divided equally across three body-weight categories (thin, average weight, and higher weight; four characters per category and gender). Each character displayed four emotional expressions: happiness, sadness, anger, and neutrality. Examples of images from this dataset appear in Fig. 3.

#### Identity preservation challenge

To maintain the same character identity across all four emotional expressions, the images containing the sad expression from Study 1 served as the source image for all expressions within a character subset. This was done since pilot testing revealed that modifying other expressions to create sadness resulted in unreliable emotional portrayals or identity shifts. As a result of this procedure to maintain identity consistency within each character subset, the resulting model identities in this study differed from those used in Study 1.

#### Advanced computational workflows

Three ComfyUI workflows were applied cyclically until validation criteria were met: (1) Expression Editor generated happiness, anger, and neutrality from the base “sad” expression; (2) LoRA-Based Inpainting with PuLID workflow (Lin et al., 2024) adjusted facial features to retain identity while ensuring that the desired expression was also maintained when expression edits caused identity deviations; (3) Reactor Node face swap refined outputs if identity similarity remained suboptimal.

#### Computational validation of identity similarity

FaceShape software (faceshape, n.d.)—a web-based tool for quantifying facial identity similarity—quantified pairwise identity similarity between images varying in emotion expressions for the same character. Benchmarking against the ADFES dataset (Van der Schalk et al., 2011) showed that same-individual images across emotions received perfect similarity scores, whereas different individuals showed significantly lower similarity (*M* = 12.57%, *SD* = 11.51%). In the final Study 2 stimulus set, all within-character emotion variants met the same-individual benchmark. Py-Feat analysis also indicated high emotion-classification confidence for the intended expressions, *M* = 97.72% (*SD* = 1.59%).

### Human validation

#### Study goals

Study 2 aimed to validate the refined AI-generated stimuli with human participants, building on Study 1’s approach while addressing its limitations through same-character methodology and incorporating anger as a fourth emotion. The validation goals paralleled Study 1: testing emotion and weight recognition, perceived realism, and perceived age ratings.

#### Participants

A total of 736 US participants (377 men, 345 women, 6 identifying as other, 8 declining to specify) with a mean age of 37 years (*SD* = 13.55) were recruited using the same criteria as Study 1.

#### Procedure and measures

The procedure followed the same approach as Study 1, with participants viewing one AI-generated image and completing validation assessments. The measures were identical to Study 1, with the addition of anger ratings to accommodate the fourth emotion condition.

### Results

#### Manipulation check validation

##### **Emotion manipulation**

**Fig. 4:**
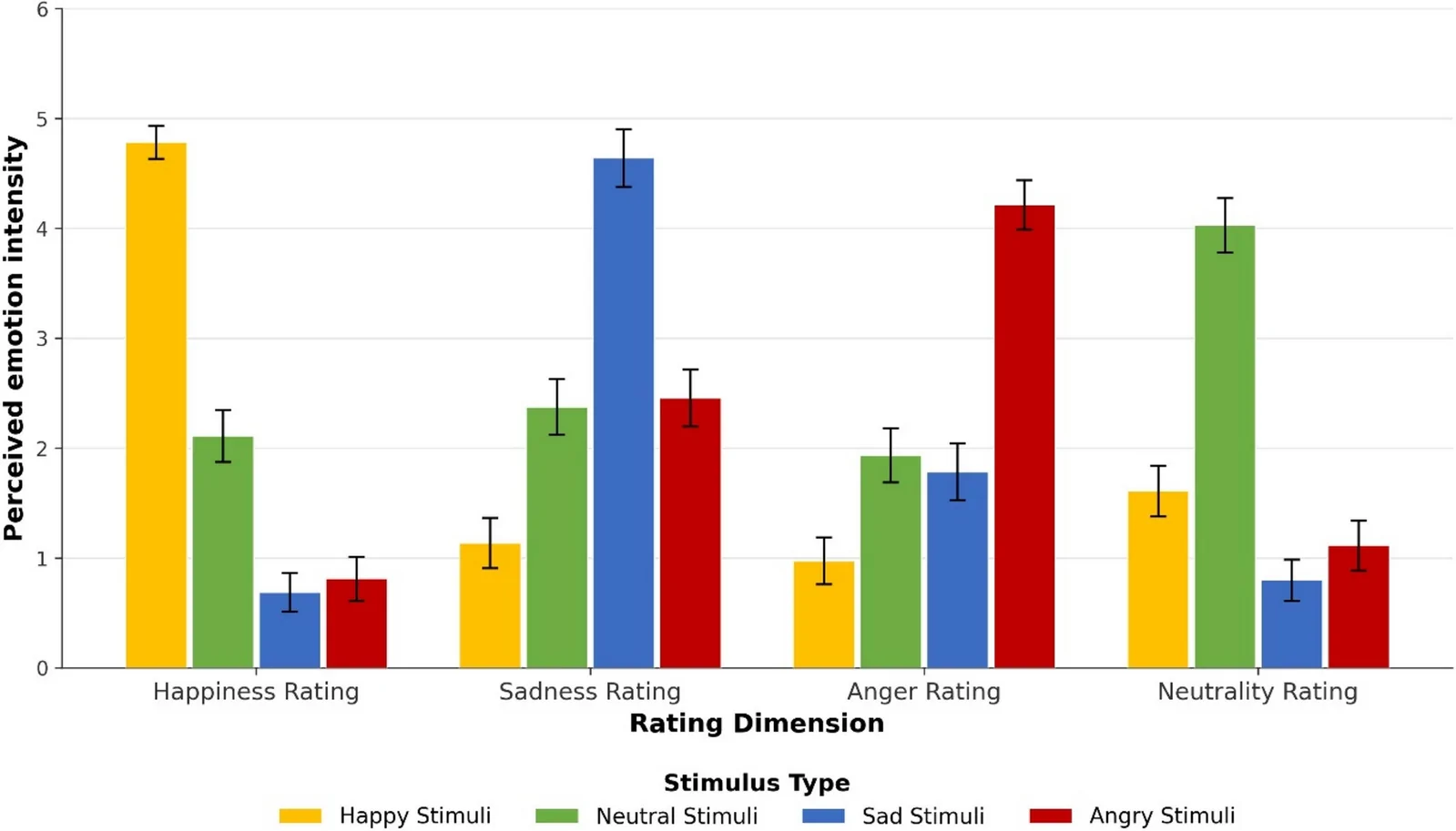
Effects of emotion expression condition on perceived happiness, sadness, anger, and neutrality: means and confidence intervals – Study 2

As in Study 1, for all emotion expression ratings, there was a significant main effect of emotion: sadness, *F*(3, 712) = 133.11, *p* < .001, ηp^2^ = .36; happiness, *F*(3, 712) = 380.47, *p* < .001, ηp^2^ = .62; anger, *F*(3, 712) = 136.73, *p* < .001, ηp^2^ = .37; and neutrality, *F*(3, 712) = 162.00, *p* < .001, ηp^2^ = .41. As shown in Fig. 4, each emotion was rated highest on its intended dimension, confirming successful emotion manipulation across all four emotions.

A main effect of gender emerged for neutrality, *F*(1, 712) = 4.66, *p* = .031, ηp2 = .01, with women (*M* = 2.00, *SD* = 2.01, 95% CI [1.80, 2.21]) rated as more neutral than men (*M* = 1.74, *SD* = 1.98, 95% CI [1.53, 1.94]). A significant gender × weight interaction emerged for happiness, *F*(2, 712) = 3.41, *p* = .033, ηp2 = .01. However, post hoc comparisons revealed no significant pairwise differences between gender–weight combinations. A three-way interaction for anger, *F*(6, 712) = 2.24, *p* = .038, ηp2 = .02, was also significant. Most importantly, anger expressions were rated significantly higher on perceived anger than all other emotions across all gender and weight combinations. Within the non-anger expressions (neutral, sad, and happy), post hoc comparisons revealed minimal differentiation, with only one significant difference: sad thin women received higher anger ratings than happy higher-weight men.

Inter-rater reliability for Study 2's emotion ratings is reported jointly with Study 2b in that study's Results section, since Study 2b reused Study 2's identical stimulus set, allowing raters to be pooled per image across the two studies for a more precise reliability estimate.

##### **Weight manipulation**

Weight manipulation was successful, *F*(2, 712) = 409.53, *p* < .001, ηp2 = .53, with clear gradual increases from thin (*M* = 163.42, *SD* = 27.71, 95% CI [160.15, 166.91]) through average-weight (*M* = 197.92, *SD* = 25.04, 95% CI [194.80, 201.04]) to higher-weight groups (*M* = 222.88, *SD* = 23.02, 95% CI [219.97, 225.80]). As in Study 1, a main effect of gender also emerged, *F*(1, 712) = 144.39, *p* < .001, ηp2 = .17, with men (*M* = 204.9, *SD* = 31.96) perceived as heavier than women (*M* = 184.5, *SD* = 35.12), replicating the pattern observed in Study 1. A significant emotion × weight interaction emerged, *F*(6, 712) = 2.17, *p* = .044, ηp2 = .02; however, post hoc comparisons showed the same weight gradient (thin < average < higher weight, all pairwise comparisons significant) within every emotion condition, with no differences between emotions at any given weight level, indicating the interaction did not reflect anything beyond the main effect of weight.

#### Stimulus quality validation

##### **Perceived realness of images**

A significant effect of weight group emerged, *F*(2, 711) = 5.19, *p* = .006, ηp2 = .014. Post hoc comparisons revealed that thin individuals were perceived as somewhat less realistic (*M* = 2.70, *SD* = 2.00; 95% CI [2.45, 2.95]) than average-weight individuals (*M* = 2.14, *SD* = 1.84; 95% CI [1.91, 2.36]). However, thin individuals did not differ significantly from higher-weight individuals (*M* = 2.41, *SD* = 2.08; 95% CI [2.15, 2.67]), and average-weight and higher-weight groups also did not differ. Critically, all group means fell below 3 on the scale, corresponding to the “Can’t decide” anchor. This suggests that, despite subtle between-group differences, participants did not clearly identify any weight group as AI-generated. Image-level realness and validation ratings for each Study 2 stimulus are reported in Table 1 in the supplementary materials on OSF, allowing readers to identify the stimuli that were perceived as most and least real.

##### **Perceived age**

Main effects were found for gender, *F*(1, 712) = 6.15, *p* = .013, ηp2 = .01, and for weight group, *F*(2, 712) = 48.29, *p* < .001, ηp2 = .12. An interaction between gender and weight group, *F*(2, 712) = 4.96, *p* = .007, ηp2 = .014, and emotion and weight group, *F*(6, 712) = 2.47, *p* = .023, ηp2 = .020, qualified these effects. Post hoc comparisons indicated that thin women (*M* = 32.47, *SD* = 8.39; 95% CI [30.98, 33.96]) appeared younger than both average-weight women (*M* = 37.82, *SD* = 9.46; 95% CI [36.17, 39.46]) and higher-weight women (*M* = 37.18, *SD* = 8.65; 95% CI [35.64, 38.72]). Similarly, thin men (*M* = 28.59, *SD* = 7.71; 95% CI [27.22, 29.95]) seemed younger than both average-weight men (*M* = 36.04, *SD* = 9.35; 95% CI [34.39, 37.69]) and higher-weight men (*M* = 38.32, *SD* = 9.75; 95% CI [36.57, 40.06]). For both genders, average-weight and higher-weight individuals were rated similarly. Thin men seemed younger than thin women. Thus, thin body weight is associated with younger age, and gender differences in age perception appear only in this thin-weight group.

Regarding the emotion × weight group interaction, for all emotion conditions except for neutrality, thin individuals were perceived as younger whereas average-weight and higher-weight individuals were perceived similarly within each emotion condition. All groups fell within what could be considered a broadly similar adult age range (late 20s to early 40s). Of most importance, these age perception patterns validate the ecological authenticity of our AI-generated stimuli. The systematic age increase across weight categories reflects documented facial adiposity-age correlations, where increased facial fat is associated with appearing older (Coetzee et al., 2010). This weight–age relationship replicates the main-effect pattern found in Study 1; unlike Study 1, however, which found no significant gender or emotion interactions with weight for perceived age, Study 2 revealed both a gender × weight and an emotion × weight interaction.

The weight–age perception relationship demonstrates that our AI generation process captured realistic perceptual relationships rather than artificial associations, increasing confidence in stimulus validity for behavioral research. While emotion–age perception patterns differed somewhat between studies, possibly due to the methodological change from different characters (Study 1) to same characters (Study 2), all stimuli across both studies fell within a similar adult age range, ensuring no problematic age confounds across experimental conditions.

### Discussion

Study 2 successfully replicated and extended the validation findings from Study 1, demonstrating that the AI generation methodology maintains effectiveness when controlling for individual facial features and incorporating additional emotions. The same-character approach eliminated potential confounds from facial feature variation while the inclusion of anger as a fourth emotion demonstrated the methodology’s versatility across a broader range of emotional expressions.

The emotion validation results confirmed that each of the four emotions was appropriately perceived, with participants correctly identifying the intended emotional expression in each condition. Computational validation showed high Py-Feat classification for the intended emotions, while FaceShape validation indicated 100% facial similarity within each character set. This dual validation approach ensured both emotional authenticity and identity consistency.

The perceived realness results paralleled Study 1, with AI-generated images maintaining photorealistic quality across conditions. Even the condition perceived as least realistic scored below the “Can’t decide” threshold, indicating that participants could not reliably distinguish AI-generated images from real photographs. Across Studies 1 and 2, weight-related differences in perceived realness were small and not fully consistent. In Study 1, higher-weight male stimuli were rated as somewhat less real than most other gender–weight combinations. In Study 2, however, the weight effect did not indicate lower realness for higher-weight stimuli: thin stimuli were rated as somewhat less realistic than average-weight stimuli, and higher-weight stimuli did not differ from either group. Importantly, even the least convincing conditions remained near the “Can’t decide” point rather than being clearly identified as AI-generated. Thus, the results suggest some sensitivity of perceived realness to body-weight cues, but not a general tendency for higher-weight stimuli to appear less real.

The weight manipulation remained effective, replicating the clear gradual increases across weight categories observed in Study 1. However, age perception effects revealed an important methodological difference between studies. While Study 1 showed a clear happy = younger main effect, this pattern did not replicate consistently in Study 2’s emotion × weight interactions. This suggests that the same-character methodology may have altered emotion-age perception dynamics, possibly because maintaining identical facial features across emotions changes how other cues influence age judgments.

## Study 2b: Additional validation of emotional and stimulus properties

The primary goal of the present research was to develop AI-generated facial-expression stimuli that are perceived as closely as possible to photographs of real human faces. Studies 1 and 2 assessed this directly by asking participants to judge whether each image depicted a real person or was AI-generated. In both studies, participants could not reliably make this distinction, with even the least convincing conditions scoring near the “Can’t decide” midpoint. The age-perception findings provided additional indirect support: perceived age varied systematically with body weight and emotional expression in ways consistent with previous findings using real human photographs, including evidence in Study 1, that happy expressions are associated with younger perceived age (Hass et al., 2016; Voelkle et al., 2012) and that greater body weight is associated with older perceived age (Coetzee et al., 2010).

Study 2b extended this validation approach by examining whether the AI-generated images also showed broader perceptual properties expected of real-person facial-expression stimuli. Facial expressions in photographs of real people are not defined only by categorical recognition or perceived intensity. They also carry broader affective information. Emotional expressions differ in perceived valence, with positive and negative emotions producing distinct evaluative impressions (Barrett & Russell, 1999; Kafetsios & Hess, 2023; Russell, 1993; Russell & Fehr, 1987; Watson et al., 1988). Neutral faces are also not perceived as emotionally empty, but may convey subtle affective or social-perceptual cues (Adams Jr. et al., 2012; Şentürk et al., 2023). Accordingly, intensity ratings were included to examine whether neutral expressions were perceived as comparatively low in expressive strength, as expected, or as entirely devoid of expression. Similarly, a facial expression typically communicates not only the focal emotion it is intended to depict, but also varying degrees of non-focal emotions (Kafetsios & Hess, 2023; Russell, 1993; Russell & Fehr, 1987). Thus, if the AI-generated images are perceived in ways similar to photographs of real people, they should show coherent patterns not only on the intended emotion category, but also on these broader affective dimensions.

Study 2b also assessed naturalness and authenticity as additional indicators of expression quality. Authenticity refers to whether an expression appears genuinely felt rather than deliberately produced, whereas naturalness refers to whether the expression looks like a typical or ordinary display of that emotion. Importantly, neither low authenticity nor low naturalness necessarily means that an image fails to appear human or real; posed expressions of real people can also appear less than fully genuine or somewhat atypical in execution. These measures are, therefore, distinct from the perceived-realness (AI-detection) measure used in Studies 1 and 2: an expression can be judged as clearly human while still appearing somewhat posed or atypical, and vice versa. This distinction is important because many facial-expression databases rely on posed rather than spontaneous expressions (Dawel et al., 2022), and perceived genuineness varies across emotions, with happiness typically perceived as more genuine than sadness or anger (Dawel et al., 2017). We expected each emotion condition to receive its highest rating on the corresponding focal emotion dimension. We also expected the emotion conditions to differ meaningfully in valence and intensity, and for naturalness and authenticity ratings to provide additional information about the perceived quality of the displayed expressions. To reduce demand characteristics, participants rated all six emotion dimensions rather than only the non-focal emotions.

### Method

#### Participants

A total of 731 participants (333 men, 392 women, 6 identifying as other) with a mean age of 40.31 years (*SD* = 12.74) were recruited via Prolific Academic using the same eligibility criteria as Studies 1 and 2. Participants were paid £0.60 for their participation, which had an estimated duration of 3 min. The study was approved under the same ethics approval as Studies 1 and 2.

#### Procedure and measures

The procedure followed that of Study 2, with each participant viewing a single AI-generated image from the Study 2 stimulus set in a between-subjects design. The study was implemented in JATOS (Just Another Tool for Online Studies; Lange et al., 2015), which allowed controlled assignment to the 24 experimental cells formed by emotion condition (neutrality, sadness, happiness, anger), weight category (thin, average weight, higher weight), and gender (female, male). Cell sizes ranged from 30 to 32 participants per cell.

Participants first rated the extent to which the person in the image expressed each of six emotions—sadness, happiness, anger, fear, disgust, and neutrality—using 7-point scales ranging from 0 (*not at all*) to 6 (*to a large extent*). Participants then rated four additional expression-related dimensions. Valence was assessed with the item, “Overall, how would you describe the emotional state of this person?” (0 = very negative, 6 = very positive). Intensity was assessed with the item, “How intense is this person’s emotional expression?” (0 = not at all intense, 6 = very intense). Naturalness was assessed with the item, “How natural does this person’s emotional expression look?” (0 = not at all natural, 6 = very natural). Authenticity was assessed with the item, “How authentic does this person’s emotional expression appear?” (0 = not at all authentic, 6 = very authentic).

### Results and discussion

Data were analyzed using 4 Emotion × 2 Gender × 3 Weight ANOVAs, following the same analytic approach used in Studies 1 and 2. Full ANOVA tables and post hoc contrast results are provided in the supplementary materials on OSF.

#### Emotion ratings

The primary validation criterion concerned the four focal emotion ratings: sadness, happiness, anger, and neutrality. This criterion was met. As shown in Table 1, each emotion condition received its highest rating on the corresponding focal emotion dimension: sadness stimuli were rated highest on sadness, happiness stimuli on happiness, anger stimuli on anger, and neutral stimuli on neutrality. Importantly, the focal ratings were not merely higher than the non-focal ratings; they were also sufficiently high in absolute terms and broadly similar to levels commonly reported in validation studies using real-person facial-expression stimuli (see, e.g., Scarantino et al., 2022). Thus, Table 1 indicates that the intended expressions were clearly perceived as such, not only statistically differentiated from the other emotion dimensions.

**Table 1:** Mean ratings (SD) [95% CI] by emotion condition for all measures: Study 2b

Measure	Neutrality	Sadness	Happiness	Anger	
	*M (SD) [95% CI]*	*M (SD) [95% CI]*	*M (SD) [95% CI]*	*M (SD) [95% CI]*	*F*(3, 707), *p* partial η2
*Emotion ratings*					
Sadness	2.73_b_ (1.54) [2.51, 2.96]	**5.13**_c_** (1.02)** **[4.98, 5.28]**	1.66_a_ (1.74) [1.41, 1.91]	2.53_b_ (1.75) [2.28, 2.79]	178.92, < .001 .43
Happiness	2.12_b_ (1.72) [1.87, 2.37]	0.48_a_ (1.00) [0.33, 0.62]	**4.58**_c_** (1.19)** **[4.41, 4.75]**	0.44_a_ (0.99) [0.30, 0.59]	440.90, < .001 .65
Anger	2.09_b_ (1.76) [1.83, 2.34]	1.54_a_ (1.54) [1.31, 1.76]	1.24_a_ (1.53) [1.02, 1.46]	**4.27**_**c**_** (1.39)** **[4.06, 4.47]**	141.11, < .001 .37
Neutrality	**4.11**_c_** (1.60)** **[3.88, 4.34]**	0.81_a_ (1.29) [0.62, 1.00]	1.71_b_ (1.50) [1.50, 1.93]	0.59_a_ (1.05) [0.44, 0.75]	252.99, < .001 .52
Disgust	2.00_b_ (1.63) [1.77, 2.24]	2.47_c_ (1.73) [2.22, 2.72]	1.22_a_ (1.60) [0.99, 1.45]	3.19_d_ (1.69) [2.94, 3.44]	45.77, < .001 .16
Fear	1.85_a_ (1.60) [1.62, 2.08]	2.33_b_ (1.70) [2.08, 2.58]	1.54_a_ (1.73) [1.29, 1.79]	1.42_a_ (1.44) [1.21, 1.63]	11.45, < .001 .05
*General emotional properties*					
Valence	2.89_b_ (0.96) [2.75, 3.03]	1.14_a_ (1.01) [1.00, 1.29]	4.61_c_ (1.16) [4.44, 4.77]	1.13_a_ (0.97) [0.99, 1.28]	485.79, < .001 .67
Intensity	2.20_a_ (1.52) [1.98, 2.42]	4.00_c_ (1.42) [3.79, 4.20]	3.07_b_ (1.48) [2.86, 3.28]	4.27_c_ (1.31) [4.08, 4.46]	80.27, < .001 .25
Naturalness	3.97_c_ (1.45) [3.76, 4.18]	2.91_b_ (1.81) [2.65, 3.18]	4.11_c_ (1.57) [3.89, 4.34]	2.22_a_ (1.61) [1.99, 2.46]	58.28, < .001 .20
Authenticity	4.01_c_ (1.39) [3.81, 4.21]	3.53_b_ (1.69) [3.28, 3.77]	4.19_c_ (1.50) [3.97, 4.41]	2.85_a_ (1.63) [2.61, 3.09]	27.21, < .001 .10

All measures on a 0–6 scale. Subscript letters indicate homogeneous subsets based on Tukey-adjusted pairwise comparisons; means sharing a letter do not differ significantly (*p* > .05). Bold values indicate the focal emotion for each stimulus condition

The neutrality ratings also clarify the status of the neutral stimuli. Neutral faces were not only rated highest on neutrality, but their ratings on the other emotion scales were not uniformly zero, indicating that they were not perceived as completely emotionless. This pattern is consistent with evidence that neutral faces can carry subtle affective meaning and may be perceived as emotionally ambiguous rather than affectively empty (Adams Jr. et al., 2012; Şentürk et al., 2023).

The main effect of emotion condition was also significant for fear and disgust ratings. Fear ratings were highest for sadness stimuli and did not differ among neutral, happiness, and anger stimuli. Disgust ratings were highest for anger stimuli, followed by sadness and neutral stimuli, and lowest for happiness stimuli. The elevated disgust ratings for anger stimuli are consistent with evidence that anger and disgust expressions are perceptually similar and are often confused in facial-expression perception (Palermo & Coltheart, 2004).

Thus, the emotion ratings supported the focal validation of the four intended expressions while also showing meaningful non-focal emotional content, consistent with evidence that facial expressions are often perceived as blends rather than pure categorical states (Kafetsios & Hess, 2023; Russell, 1993; Russell & Fehr, 1987).

##### Inter-rater reliability of perceived focal emotions (Studies 2 and 2b)

Because Study 2b reused the exact 96 images from Study 2, ratings were pooled across the two data collections to assess inter-rater reliability, following the same one-way random-effects intraclass correlation approach used in Study 1 (ICC(1,k); Shrout & Fleiss, 1979). Pooling raters across the two data collections yielded good reliability on the focal emotion rating, ICC(1,k) = .626, 95% CI [.445, .722] (Cicchetti, 1994), based on an average of 15.28 raters per image. Per-study ICC estimates, per-image rater counts, and the homogeneity check are reported in the OSF supplementary materials.

#### Gender and body-weight patterns in emotion ratings

Gender and body weight also shaped some emotion ratings. These effects are relevant because images of real people are not perceived only in terms of the expression displayed; social-category information can also contribute to emotion perception (Hess et al., 2009).

Anger ratings showed a significant main effect of gender, *F*(1, 707) = 7.84, *p* = .005, η2 = .011, with male stimuli rated as angrier (*M* = 2.43, *SD* = 2.00, 95% CI [2.23, 2.64]) than female stimuli (*M* = 2.12, *SD* = 1.90, 95% CI [1.92, 2.31]). A gender × weight interaction also emerged, *F*(2, 707) = 3.22, *p* = .040, η2 = .01, but follow-up comparisons did not identify reliable pairwise differences. The overall gender effect is consistent with evidence from studies using real faces that anger is more strongly associated with male than female targets (Hess et al., 2004).

Sadness ratings showed a significant main effect of weight, *F*(2, 707) = 8.81, *p* < .001, η2 = .024. This effect was qualified by an emotion × weight interaction, *F*(6, 707) = 2.68, *p* = .014, η2 = .022, and an emotion × gender × weight interaction, *F*(6, 707) = 2.95, *p* = .007, η2 = .024. Follow-up analyses showed that these interactions did not reflect a failure of the sadness manipulation: sadness stimuli were rated as highly sad across all gender × weight combinations, with means ranging from 4.70 to 5.33. Rather, the interactions reflected variation in the extent to which non-sad expressions were also perceived as somewhat sad across weight and gender conditions. Thus, body weight and gender contributed to sadness ratings, but they did not compromise the focal validation of the sadness expressions. Full cell means and post hoc comparisons are reported in the Markdown of Study 2 in the supplementary materials on OSF.

Fear ratings also showed a significant main effect of weight, *F*(2, 707) = 5.17, *p* = .006, η2 = .014. Higher-weight stimuli were rated as more fearful (*M* = 2.05, *SD* = 1.67, 95% CI [1.84, 2.26]) than both thin stimuli (*M* = 1.60, *SD* = 1.63, 95% CI [1.39, 1.80]) and average-weight stimuli (*M* = 1.70, *SD* = 1.65, 95% CI [1.50, 1.91]), which did not differ from each other. Disgust ratings showed a significant main effect of gender, *F*(1, 707) = 7.67, *p* = .006, η2 = .011, with male stimuli rated higher on disgust (*M* = 2.38, *SD* = 1.83, 95% CI [2.19, 2.57]) than female stimuli (*M* = 2.04, *SD* = 1.78, 95% CI [1.86, 2.23]).

Taken together, these gender- and body-weight-related effects suggest that the stimuli were not perceived as abstract emotion displays stripped of social information. Rather, gender and body weight contributed to some emotion judgments. The gender effect on anger is consistent with prior evidence that anger is more strongly associated with male than female targets, whereas the weight-related effects show that body-weight cues also entered into emotion perception. Importantly, these effects did not alter the focal validation result: each intended emotion condition was rated highest on its corresponding focal emotion dimension.

### Valence and intensity

Valence ratings showed a significant main effect of emotion condition. As shown in Table 1, happiness stimuli were rated as positive, sadness and anger stimuli as negative, and neutral stimuli near the midpoint of the scale. This pattern is consistent with dimensional accounts of affect, in which emotional expressions differ not only categorically but also in their positive or negative evaluative meaning (Barrett & Russell, 1999; Watson et al., 1988).

To further characterize the neutral stimuli, neutral-valence ratings were tested against the scale midpoint using a one-sample *t* test. Because each participant viewed a single image from one of the four emotion conditions, tests restricted to a specific emotion are based on the subset of participants assigned to that condition (n ≈ 184 for neutral), rather than the full Study 2b sample (N = 731). Neutral stimuli did not differ significantly from the midpoint, *t*(183) = −1.53, *p* = .128, indicating that they were affectively neutral on average. Because neutral faces may nevertheless show small deviations from neutrality, we also examined the neutral stimuli at the image level. Mean valence ratings were computed separately for each of the 24 neutral images. These image-level means ranged from 2.13 to 3.38 on the 0–6 scale, with an average image mean of 2.88 and an *SD* of 0.33, indicating modest descriptive variation around the midpoint. This degree of variation is compatible with validation work on neutral real-person face databases, where valence deviations are also interpreted within a highly neutral stimulus set rather than as evidence of strong positive or negative expression (Şentürk et al., 2023). Given the small number of ratings per image, this analysis should be interpreted descriptively. Full stimulus-level descriptives and exploratory inferential analyses are reported in the supplementary materials on OSF.

Valence also showed a significant main effect of gender, *F*(1, 707) = 8.69, *p* = .003, η2 = .012, with female stimuli rated as slightly less negative (*M* = 2.56, *SD* = 1.75, 95% CI [2.38, 2.74]) than male stimuli (*M* = 2.35, *SD* = 1.78, 95% CI [2.17, 2.53]). Although small, this effect is consistent with evidence that face gender can bias affective impressions, such that female faces are associated with relatively more positive evaluations than male faces (Becker et al., 2007).

Intensity ratings showed a significant main effect of emotion condition. As shown in Table 1, anger and sadness stimuli were rated as most intense, happiness stimuli as moderately intense, and neutral stimuli as least intense. Thus, perceived expressive strength varied systematically across emotion conditions. Importantly, neutral expressions were not rated as entirely devoid of intensity: their mean intensity rating was significantly above zero, *M*=2.20, *SD*=1.52, *t*(183) = 19.63, *p* < .001. Intensity also showed a significant main effect of gender, *F*(1, 707) = 6.83, *p* = .009, η2 = .010, with male stimuli rated as slightly more intense (*M* = 3.51, *SD* = 1.64, 95% CI [3.34, 3.68]) than female stimuli (*M* = 3.25, *SD* = 1.65, 95% CI [3.08, 3.41]). Thus, the intensity results indicate systematic variation in perceived expressive strength, with neutral expressions comparatively low but not entirely absent in intensity.

### Naturalness and authenticity

Significant main effects of emotion emerged for both naturalness and authenticity. As shown in Table 1, the ordering was highly similar across the two measures: happiness and neutral expressions were rated as most natural and authentic, sadness expressions received intermediate ratings, and anger expressions received the lowest ratings. One-sample *t*-tests against the scale midpoint were conducted for authenticity because this measure directly indexed whether expressions appeared genuinely felt rather than merely more or less authentic relative to one another. These tests showed that happiness, neutral, and sadness expressions were rated as significantly more authentic than the midpoint, *t*(184) = 10.78, *p* < .001, *t*(183) = 9.86, *p* < .001, and *t*(181) = 4.21, *p* < .001, respectively. By contrast, anger did not differ from the midpoint, *t*(179) = −1.23, *p* = .219, indicating that anger expressions were perceived as neither clearly authentic nor clearly inauthentic.

This ordering—happiness ≈ neutral > sadness > anger for both naturalness and authenticity—is consistent with the genuineness hierarchy established for posed expressions in commonly used facial expression databases, where happiness is perceived as most genuine, sadness as intermediate, and anger as least genuine (Dawel et al., 2017). A significant main effect of gender also emerged for naturalness, *F*(1, 707) = 9.38, *p* = .002, η2 = .013, with female expressions rated as more natural (*M* = 3.49, *SD* = 1.78, 95% CI [3.31, 3.68]) than male expressions (*M* = 3.14, *SD* = 1.78, 95% CI [2.95, 3.32]).

Study 2b extended the validation beyond categorical emotion recognition. The four intended expressions were clearly perceived, with focal ratings broadly comparable to levels reported in prior validation studies using real-person facial-expression stimuli. The additional ratings showed coherent affective and expression-quality profiles: the stimuli carried meaningful non-focal emotional content, showed expected valence differences, varied systematically in expressive intensity, and differed in naturalness and authenticity in ways consistent with posed-expression research. Neutral stimuli were perceived as neutral and comparatively low in intensity, but not as affectively or expressively empty. Anger was the weakest condition in naturalness and authenticity, consistent with evidence that posed anger expressions are often perceived as less genuine than happiness or sadness. Overall, Study 2b supports the use of the stimuli as controlled AI-generated images that approximate key perceptual properties of real-person facial-expression stimuli.

## General discussion

This research presents a comprehensive creation and validation framework for AI-generated facial expression stimuli in psychological research, demonstrating that current AI technology can produce research-quality stimuli that meet rigorous scientific standards. Across three studies involving over 2,000 participants, we established that AI-generated facial expressions effectively convey emotional information, enable systematic manipulation of physical characteristics, and achieve photorealistic quality indistinguishable by humans from real photographs. These conclusions align with emerging research demonstrating that contemporary AI systems can produce faces difficult for human observers to distinguish from real photographs (Miller et al., 2023; Nightingale & Farid, 2022) and that AI-generated facial expressions can, in addition, effectively convey emotional information comparable to photographed expressions (Han et al., 2022).

### Methodological contributions

The dual validation approach combining computational and human assessment provides a robust framework for ensuring AI-generated stimulus quality. Computational validation using Py-Feat analysis achieved over 95% classification confidence for the intended emotion in both studies, while human validation confirmed appropriate perception across all target dimensions. This convergent validation approach addresses concerns about AI-generated stimuli by demonstrating effectiveness through multiple assessment methods.

The systematic manipulation of body weight alongside emotional expressions demonstrates AI technology’s potential for addressing methodological challenges in social psychology research. Traditional approaches to studying weight bias have been constrained by ethical considerations and limited stimulus availability (Ruggs et al., 2010). AI-generated stimuli circumvent these limitations while enabling precise experimental control.

The same-character methodology introduced in Study 2 represents an important advance for controlling individual facial feature variation. By maintaining identical facial characteristics across different emotional expressions, this approach enables researchers to isolate effects of target manipulations while controlling for potential confounds.

Critically, the validation results suggested that the stimuli captured several realistic perceptual relationships rather than merely satisfying the intended manipulations. Study 2b extended this evidence to broader affective and expression-quality dimensions. For example, happiness was rated as positive, sadness and anger as negative, neutral expressions were affectively neutral on average but not expressively empty, and naturalness/authenticity varied across emotions in a pattern consistent with posed real-person facial-expression databases. Together, these findings support the ecological validity of the stimuli by showing that observers responded to them in psychologically meaningful ways, not only by identifying the intended categories.

#### Implications for emotion perception research

The successful validation of AI-generated emotional expressions has significant implications for emotion perception research and social perception more generally. These stimuli offer advantages over traditional approaches including: precise control over multiple attributes simultaneously, elimination of privacy and consent concerns, ability to generate large numbers of exemplars, and systematic manipulation of characteristics difficult to control in photographed stimuli.

The methodology enables investigation of previously challenging research questions, particularly those involving stigmatized characteristics or sensitive topics. Research on weight bias, for example, can now employ systematically controlled stimuli without ethical concerns about using real individuals’ images.

The present studies prioritized unambiguous emotion-category membership rather than subtle, ambiguous, or mixed emotional displays. Computational screening therefore required each image to reach at least 95% Py-Feat likelihood for the intended emotion category. However, everyday emotion perception often involves expressions that are less prototypical, more context-dependent, and open to multiple interpretations (Barrett et al., 2019; Lange et al., 2022). The same general framework could therefore be extended to create stimuli depicting mixed or ambiguous emotional expressions, for example, expressions combining anger and disgust, sadness and fear, or neutrality with subtle affective cues. Such stimuli would require a different validation strategy, focusing not only on whether a single intended category is recognized, but also on the full profile of emotion ratings, perceived valence, intensity, naturalness, and authenticity. This approach offers flexibility, as LoRAs can be trained with datasets representing different types of emotion expressions, such as spontaneous rather than posed expressions or of mixed emotions. The validity of the resulting stimuli can then be assessed with Py-Feat by comparing the analysis results against a standard derived from running the same analysis on the original dataset used to train the LoRA. Importantly, this capability extends beyond facial expressions to other visual aspects that researchers wish to control in their stimuli. These may include features such as ethnicity, age, specific facial characteristics representing underrepresented identities, or other attributes that require precise control. Multiple LoRAs can be combined within a single workflow to control several features concurrently, such as pairing facial expression LoRAs with ethnicity LoRAs to achieve precise multidimensional stimulus control.

#### Limitations and future directions

Although participants generally perceived the stimuli as real, and several secondary validation patterns aligned with findings from real-person image research, these results do not imply that AI-generated and real-world facial-expression stimuli are equivalent in all respects. Subtle differences may remain in aspects not captured by the present validation measures, such as skin texture, color saturation, micro-expressions, or other perceptual cues that observers may not report explicitly. This possibility is consistent with research showing that while current AI-generated faces have largely “crossed the uncanny valley” for static images (Nightingale & Farid, 2022), computational algorithms can still detect subtle artifacts that humans cannot consciously perceive (Farid, 2022). Thus, the stimuli can be treated as photorealistic and psychologically meaningful research materials, but not as complete substitutes for real-world facial expressions in every context. As image-generation models continue to improve, this gap may narrow, but direct validation against human perception will remain essential.

The current validation focused on static facial expressions. Dynamic facial expressions have been shown to provide advantages over static displays in emotion recognition, particularly for more naturalistic emotion perception (Krumhuber et al., 2013; Richoz et al., 2018). Future research should explore video-based AI-generated expressions and temporal dynamics of emotional displays to capture the full richness of natural emotional communication. Although the present framework focused on static images, the same logic could be extended to dynamic facial-expression stimuli. This extension would require additional validation steps beyond those used here. In particular, video-based generation must preserve identity consistency across frames, avoid temporal artifacts, and control the temporal unfolding of the expression, including onset, apex, and offset. Within a ComfyUI-based workflow, tools for image-to-video or animation-based generation could be combined with the same identity-preservation and expression-validation principles used here, but validation would need to occur at both the frame level and the clip level. Researchers would need to assess not only whether the intended expression is recognized, but also whether the transition appears temporally natural and whether the same identity is preserved throughout the sequence.

Integration with other modalities such as vocal expressions could further enhance stimulus realism and research applications. Multimodal emotion recognition research shows that combining facial expressions with vocal cues provides more comprehensive emotional information than either modality alone (Zhang et al., 2020). As AI technology advances in speech synthesis, future validation frameworks could extend to encompass audiovisual stimuli that maintain the same level of experimental control demonstrated here for facial expressions.

A practical limitation of the present framework is that, although the tools are open source, the full pipeline still requires substantial technical familiarity. Researchers need to manage ComfyUI workflows, LoRA training, identity-preservation procedures, image refinement, and computational validation. Thus, the method is more accessible than bespoke 3D modeling or commercial animation pipelines, but it is not yet a simple point-and-click solution. A useful future direction would be to package the workflow into template pipelines or graphical interfaces in which researchers can specify the desired emotion, identity constraints, and validation thresholds without directly managing all workflow components.

### Ethical considerations

The use of AI-generated stimuli addresses several ethical concerns in psychological research while introducing new considerations. AI-generated faces eliminate privacy concerns and consent complications associated with real individuals’ photographs. However, researchers must consider potential biases in AI training data and ensure generated stimuli represent diverse populations appropriately.

## Conclusion

This research demonstrates that AI-generated facial expression stimuli can serve as a viable and advantageous alternative to traditional photographed stimuli in psychological research. The comprehensive dual validation framework offers researchers enhanced control over stimulus characteristics while maintaining ecological validity and addressing ethical considerations inherent in using real individuals’ photographs.

The successful development and validation of this AI generation methodology opens new possibilities for psychological research, particularly in areas requiring systematic stimulus control or involving sensitive characteristics. As AI technology continues advancing, this framework provides a foundation for ensuring research-quality stimuli that meet scientific standards while leveraging technological capabilities for enhanced experimental precision.

Importantly, the approach is not limited to the exact tools employed in this research. The validation framework principles can be applied with other AI generation tools beyond ComfyUI and Flux1-dev and different validation tools beyond Py-Feat, maintaining the same methodological rigor. This flexibility ensures the framework’s continued relevance as AI technologies evolve.

Future research should continue developing and refining AI-generated stimulus methodologies while expanding applications across diverse populations and research contexts. The convergence of psychological research needs with advancing AI capabilities promises continued innovation in experimental methodology and enhanced understanding of human perception and cognition. The dual validation approach established here provides a template for ensuring that technological advances translate into scientifically rigorous research tools.

## Footnotes

1 The body weight descriptors in prompts used the terms “fit,” “middle weight,” and “obese,” as these terms were optimized for the base model's training vocabulary and produced more reliable generation results. Throughout this paper, we use the more neutral terminology “thin,” “average weight,” and “higher weight” to describe the same categories in a less stigmatizing manner.

## Declarations

### Open practices statement

All validation data, participant responses, generated stimulus datasets, and supplementary materials are openly available via OSF at: https://osf.io/mbg4j/?view_only=a0c0512fa2b04cc19c335cc2bd299017. This includes complete raw datasets for all studies, data codebooks, all AI-generated stimuli, PyFeat and FaceShape analysis results, complete analysis reports, and supplementary materials.

All computational code and workflows are openly available in the same OSF repository, including R Markdown files, Python scripts, ComfyUI workflow specifications (.json files), LoRA models (.safetensors files), and step-by-step instructions.
